# The Surgery of Malignant Disease

**Published:** 1914-02-28

**Authors:** 


### MODERN METHODS SERIES.

### The Surgery of Malignant Disease.

Surgeons have for a long time been telling the public in general and the medical profession in especial that their results in the operative treatment of malignant disease would be vastly improved if only they saw the cases earlier. This involves problems of diagnosis which are in many cases more difficult than they sound. With the best will in the world, the general practitioner may have the greatest hesitation in making up his mind that some minor symptoms of indigestion, let us say, are really due to malignant disease of the stomach; or even that they are sufficiently suspicious to make further investigation, which is apt to be a somewhat costly affair, imperative. Even the surgeon and the regional specialist are frequently uncertain of diagnosis in the early stages of disease; and the general practitioner sees cases, broadly speaking, at a yet earlier date in their clinical course. Still, it is true that the less thorough and conscientious type of general practitioner does fail to diagnose malignant disease sometimes when a more painstaking colleague might be able to do so, and in consequence fails to do his duty by his patients.

Another obstacle, and an even greater one, to the very early diagnosis of malignant tumours, is the reluctance which many patients display to take medical advice. The attitude too often adopted is, "If I *have* got cancer, I would much rather not know it;" and so advice is not sought until it is too late, or at any rate until the chance of complete cure is seriously minimised. This mental aberration arises, no doubt, from the persistence of the old idea that malignant disease is necessarily incurable and fatal. If only people could be induced to understand what a splendid chance of complete permanent cure is offered by surgery, *provided it is resorted to at the outset*, no one in his senses would put off investigation of any lump, tumour, or growth of any kind.

### The Pigmented Mole.

In the *American Journal of the Medical Sciences* Dr. J. C. Bloodgood brings forward a mass of clinical evidence to prove that early surgery does genuinely provide good prospects of cure. He takes up several common sites of malignant disease and several kinds of "precancerous" conditions there, and quotes the statistics of the Johns Hopkins Hospital in connection with them.

The pigmented mole is a form of benign tumour which affects a large proportion of the population. Very occasionally such a mole will develop into a melanotic sarcoma, one of the most deadly cancers known. Dr. Bloodgood found that of sixty-eight such cancers forty-three developed in a mole present from birth, and the other twenty-five in a mole formed in after-life. Only one of the patients was saved permanently. In the same period of time 175 pigmented moles were removed as a precautionary measure: there has not been a single recurrence in this series, nor any development of malignancy. Some moles, says the author, are more dangerous—that is, more likely to develop malignancy—than others; but Dr. Bloodgood would remove them all so as to be on the safe side.

Again, most connective-tissue tumours are benign —that is, non-cancerous. Yet it is so exceedingly difficult to distinguish between such an innocent growth and the early stage of a malignant one that he always advises immediate operation. Moreover, he finds that about 25 per cent. of them are actually malignant.

Of epithelial growths the proportion which are innocent is much smaller: in fact it is but 17 per cent., compared with 83 per cent. cancerous. Warts, areas of keratosis, and benign ulcers have been treated in 173 cases at the Johns Hopkins Hospital without a single local recurrence or death. Over 800 cases of epithelioma of the lips, face, eyelid, chin, ear, nose, scalp, mouth, tongue, skin, and surface of the body generally have been admitted; and it is stated that in every single case the growth had developed on a pre-existing "innocent" lesion. The percentage of cures in this class of cancer varies from 20 to 98 in the different miscroscopical varieties.

### A Cancer Dogma.

As to cancer of the breast, Dr. Bloodgood formulates the following dogma. If every woman over twenty-five years of age were to seek surgical advice the moment she felt a lump in the breast, and the surgeon explored this lump at once, the probabilities are that it would be benign (non-cancerous) in 33 per cent. of cases. Of the malignant cases, about 20 per cent. would be adenocarcinoma, with a chance of cure of almost 100 per cent.; of the other malignant types of growth the chance of cure (in the very early stage) would be about 85 per cent.

